# *De Novo* Proteins Template the Formation of Semiconductor Quantum Dots

**DOI:** 10.1021/acscentsci.4c01826

**Published:** 2025-05-27

**Authors:** Yueyu Yao, Jingyun Wu, Yue Hu, Laura Haubold, Obinna Uzosike, Guangming Cheng, Nan Yao, Gregory D. Scholes, Michael H. Hecht, Leah C. Spangler

**Affiliations:** †Department of Chemistry, ‡Department of Molecular Biology, §Princeton Materials Institute, 6740 Princeton University, Princeton, New Jersey 08544, United States; ∥ Department of Chemical and Life Science Engineering, 6889 Virginia Commonwealth University, Richmond, Virginia 23284, United States

## Abstract

Here, we present the first instance of utilizing *de novo* proteins to regulate the size of cadmium sulfide (CdS) quantum dots. Four proteins were found to bind to CdS and cap the growth of CdS quantum dots, leading to precise size control, as evidenced by absorbance and fluorescence spectra. Increasing the concentration of CdS does not change the absorbance and emission peaks, thereby indicating that the proteins effectively constrain the size of the quantum dots. Employing different proteins also yielded quantum dots with distinct optical and physical properties, including the appearance of biomediated nanorods when SynI3 was utilized. Moreover, the *de novo* proteins effectively maintained the stability of the quantum dots for up to 7 days, surpassing the stability of quantum dots capped by the small molecule, l-cysteine. The ability to cap CdS likely stems from their affinities for Cd^2+^, yet there does not seem to be a direct correlation between the affinity for Cd^2+^ and the size of resulting quantum dots.

## Graphical abstract

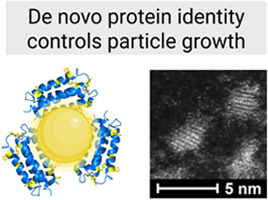

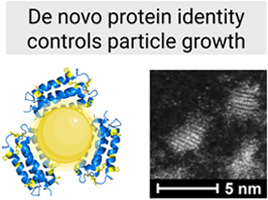

## Introduction

Protein-mediated biosynthesis has significant potential as a sustainable approach for producing inorganic nanomaterials for applications in fields ranging from healthcare to energy production.^1,2^ While it is appealing to adapt naturally evolved proteins to produce technologically relevant biomaterials, such approaches are currently limited to specific types of materials that preexist in nature.

As new technologies develop for the biogenic fabrication of non-natural materials, it is important to consider the suitability of the proteins used in these efforts: Natural proteins arose in response to eons of evolutionary selection for macromolecules that perform life sustaining tasks in specified chemical and biological environments. Therefore, natural examples of protein-mediated biosynthesis typically produce inorganic materials that are used for structural support and/or defense of the host organism, with examples including bones and shells.^3^ Moreover, the inorganic materials produced by natural proteins are typically synthesized from precursors that are abundant in natural environments. Because these functions were honed by eons of natural selection, proteins isolated from biological systems carry ‘evolutionary baggage,’ which may constrain their suitability for novel nonbiological applications.

To broaden the potential of protein-mediated biosynthesis of inorganic nanomaterials, we have initiated a program that uses proteins that did *not* evolve in nature. Instead, we use *de novo* proteins with amino acid sequences designed in the laboratory. Furthermore, to explore beyond the limitations imposed by individual sequences, we probe the biosynthetic capabilities of many different proteins isolated from large combinatorial libraries of *de novo* sequences.

To facilitate this exploration, we have designed and constructed libraries of novel amino acid sequences.^4^ Since random sequences rarely fold into ordered structures, our libraries are not fully random, but are constrained by binary patterning of polar and nonpolar amino acids that direct hydrophobic residues toward the protein interior and hydrophilic residues toward the aqueous surface.^4^ Over the years, we have generated both β-sheet libraries and α-helical libraries.^5,6^ Proteins from the β-sheet libraries were shown to form biomaterials in which assembly was templated by highly ordered pyrolytic graphite surfaces.^6^ In contrast, proteins from our α-helical libraries were shown to perform a range of biologically important functions.^7–9^

Recently, we demonstrated that one of our *de novo* α-helical proteins – called ConK – catalyzes the synthesis of semiconductor quantum dots.^10^ Specifically, ConK catalyzes the formation of CdS nanoparticles by generating reactive sulfur precursors through the desulfurization of the amino acid cysteine. The resulting H_2_S reacts with Cd acetate to form CdS nanocrystals.^10^ This discovery showed that proteins that did not evolve in nature can produce nonbiological semiconductor nanomaterials, with potential uses in a range of applications from photovoltaics to photocatalysis. Instead of relying on nature to evolve proteins for such materials, which may never happen, ConK showed that *de novo* proteins can be specifically targeted to produce such materials.

The function of ConK in producing CdS nanocrystals is catalytic: The protein, together with the cofactor PLP (pyridoxal phosphate), catalyzes the breakdown of cysteine to form pyruvate, ammonia, and H_2_S, with the H_2_S serving as a reagent for quantum dot synthesis. In principle, however, proteins involved in biomineralization can function in two ways: They can (i) catalyze the formation of reactive precursors for nanomaterial synthesis (as does ConK), or (ii) template or cap mineral crystallization.^11^ Indeed, in nature, the templating function is more commonly observed, and is used to control the final morphology and shape of biomaterials by directly binding to the material surface during nucleation and growth.^12–14^ Several examples include the silica skeleton of sponges, the magnetic particles in magnetotactic bacteria, and the intricate silica skeletons of diatoms.^15,16^

While ConK opened the possibility of utilizing novel proteins for the biogenic synthesis of optoelectronic nanoparticles by advancing the *catalytic* route, *de novo* proteins have yet to be used to *template* nanoparticles. Therefore, the current work asks whether novel proteins can also template or cap the formation of semiconductor quantum dots. Because our novel proteins are derived from combinatorial libraries of binary patterned sequences designed to fold into a family of similar 4-helix bundle structures, we can readily test whether related proteins with related structures, but different sequences can template the formation of quantum dots with different sizes and morphologies.

The impact of amino acid sequence on nanoparticle formation was previously studied using short chain peptides (6–20 amino acids). Looking toward improving control over the final material property, Belcher et al. demonstrated that short peptide sequences can be used to mineralize CdS, ZnS, and PbS as both spherical nanocrystals and nanorods.^17,18^ Importantly, these experiments found that both the amino acid residues AND the conformation of the peptide, either linear or constrained, changed the effectiveness of the peptide binding to the nanomaterial surface and thus final material shape and size. The importance of peptide conformation suggests that the arrangement of the binding amino acids, not just their identity, plays a role in controlling material growth and can be used to improve nanoparticle synthesis. This same concept is applied to our *de novo* proteins, which are much longer and more structurally complex than short chain peptides, thus offering a potentially higher level of control and more diversity in final material architecture.

The results described herein demonstrate that *de novo* proteins with a high number of metal binding residues can indeed template the formation of CdS quantum dots from an aqueous phase reaction. The resulting CdS nanocrystals grow to a uniform size in the quantum-confined region and show no further growth even with the addition of more reactive precursors. The size and shape of the resulting nanocrystals depend on which of our novel proteins is used; and in one case, a *de novo* protein led to the formation of CdS nanorods. Importantly, CdS nanocrystals capped by *de novo* proteins have higher stability than those capped with the amino acid cysteine, demonstrating the importance of the protein structure for binding to the material surface and controlling nanoparticle stability. Overall, this work demonstrates that *de novo* proteins can be used to control both nanocrystal size and overall morphology of nonbiological materials, and that modulating amino acid sequence can potentially lead to improved material properties. Taken with our previous work on ConK, the current findings demonstrate that novel proteins that did not evolve in nature can be used to both catalyze and template the formation of inorganic semiconductor quantum dots.

## Results and Discussion

In previous work, we screened a binary-patterned library of *de novo* proteins for binding to Zn^2+^, Co^2+^, or Cu^2+^ and identified 52 Naïve Metal Binders (NMBs).^9^ Due to their unevolved nature, these proteins showed promiscuous binding to several different metals. Based on this observed promiscuity, we hypothesized that the NMB proteins might also bind cadmium and thereby control the size of CdS quantum dots. Therefore, we chose two well-expressed proteins from this collection, NMB20 and NMB25, for testing as potential capping agents.

Due to design considerations in previous studies, none of the NMB proteins contain cysteine, a residue common in the metal binding sites of natural proteins. Therefore, we also constructed S824-GGC, by installing Gly-Gly-Cys at the C-terminus of protein S824, a well-expressed *de novo* sequence with a 4-helix bundle structure determined previously by NMR.^5^ Although the parental protein, S824, does not exhibit strong metal-binding, we hypothesized that S824-GGC might interact with Cd^2+^ and cap CdS quantum dots.

A fourth protein, SynI3, was included due to its previously observed catalytic activity in producing H_2_S. In the current study, we investigated the capping ability of SynI3. In future studies, we plan to explore the possibility of a single protein performing both functions by converting cysteine to H_2_S and also capping the growing CdS quantum dots, thereby leading to the complete synthesis of quantum dots driven by a single *de novo* protein.

The sequences of the four proteins described above are shown in Figure 1a. As expected from their design, all of them were predicted by AlphaFold^19^ to fold into 4-helix bundles (Figure 1 **b–e**). Experimental characterization by NMR also showed that protein S824 folds into a 4-helix bundle.^5^

**Figure 1:**
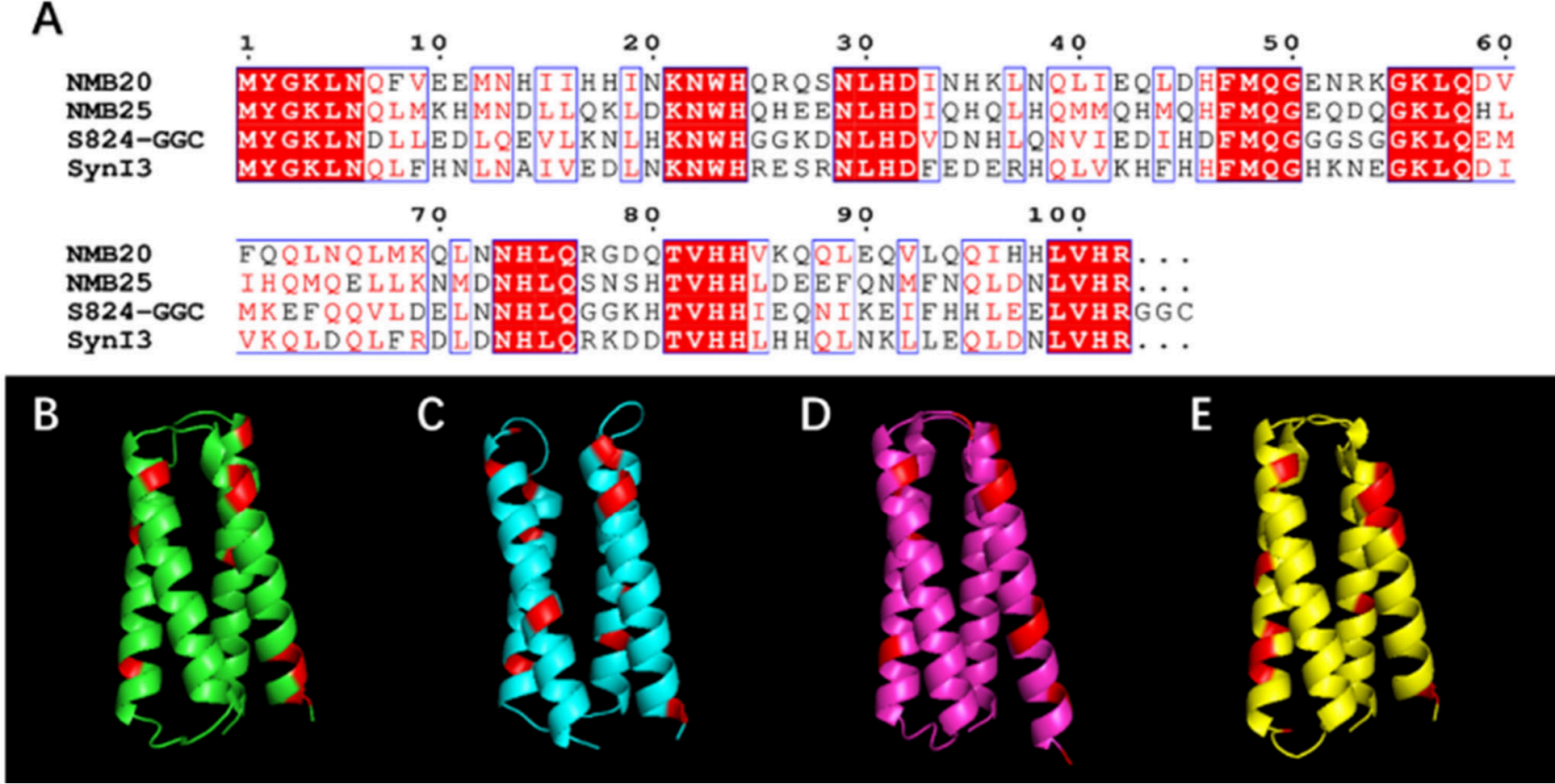
***De novo* protein-based quantum dot capping agents. (A)** Sequences of four *de novo* proteins employed as capping agents. Conserved residues from the design are highlighted in red, while regions containing different residues with shared polarity or nonpolarity are shown in black and red font. **(B-E)** Structures predicted by AlphaFold for (B) NMB20, (C) NMB25, (D) S824-GGC, and (E) SynI3. Potential metal-binding side chains (His and Cys) are highlighted.

To examine whether a *de novo* protein can cap CdS quantum dots, we compared the absorbance spectra of CdS particles synthesized in the presence of a protein relative to those of particles synthesized with l-cysteine as a capping agent or in buffer without any capping agent. The method began by premixing each capping agent with reactive sulfur precursors (NaSH) in buffer. After thorough mixing, the cadmium precursor solution was titrated into this solution, thereby initiating the reaction with sulfur to form CdS. After each addition of Cd^2+^, the solution was mixed vigorously and incubated at room temperature for 15 min. Since the average size of quantum dots is correlated with the wavelength of peak absorbance,^20^ we used absorbance to monitor the size of the newly synthesized nanoparticles. If a *de novo* protein caps the growth of a CdS quantum dot and thereby restricts its size, the peak wavelength will remain constant through successive additions of Cd^2+^. Meanwhile, the intensity of absorbance at that wavelength can be used to follow the increasing concentration of nanoparticles following each addition of Cd^2+^.

Figure 2a shows the absorbance spectra of reaction mixtures containing protein NMB25 (0.5 mg/mL) and NaSH (0.5 mM), with cadmium acetate introduced in small increments (0.1 mM per addition). After the first addition of Cd^2+^, an absorbance peak at 340 nm appeared, corresponding to size-confined CdS quantum dots ∼2 nm in diameter. The magnitude of peak increased after each addition of Cd^2+^, but the wavelength did not change, indicating that despite increasing concentration of CdS, the size of the quantum dots remained constant.

**Figure 2:**
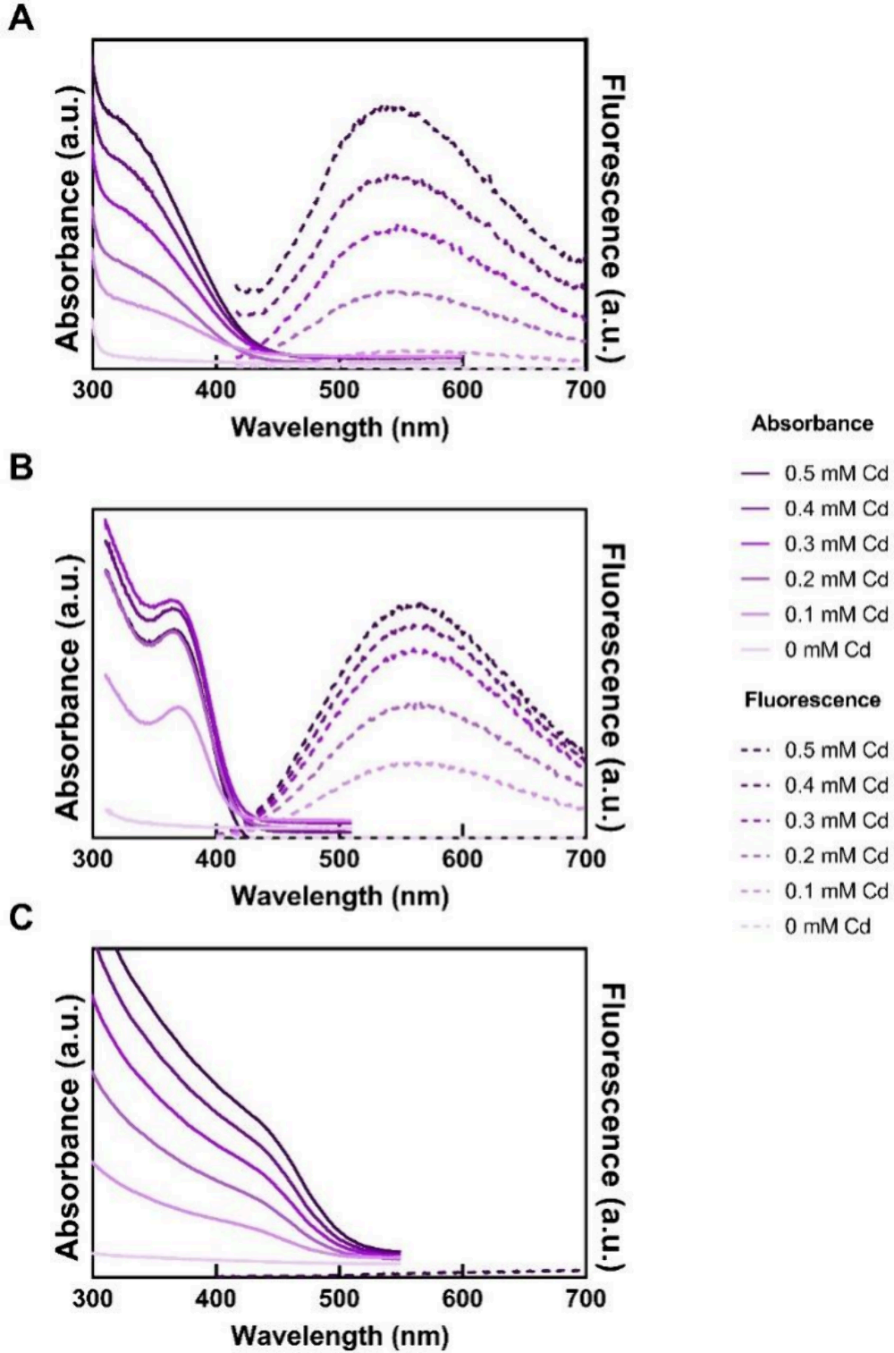
Absorbance and fluorescence spectra of CdS quantum dots formed with (a) Protein NMB25, (b) l-cysteine, and (c) no capping agent. Protein NMB25 and l-cysteine control particle size despite the increasing concentration of cadmium precursor.

We also monitored the fluorescence emission of the reaction mixture (Figure 2a). Again, the intensity of the fluorescence increased with addition of Cd^2+^, but the peak wavelength remained constant at 540 nm, demonstrating that the size of the quantum dots did not change. In contrast, in controls without capping, CdS quantum dots continued to grow (see below), and fluorescence diminished when the particles grew to sizes that were no longer quantum-confined. Thus, our results using both absorbance and fluorescence indicate that protein NMB25 restrains the size of CdS quantum dots.

To put these results in context, we compared them to capping by the small molecule, l-cysteine, and to particle growth in the absence of any capping agent. As shown in Figure 2b, adding l-cysteine produced absorbance peaks at 360 nm, which did not shift as the concentration of Cd^2+^ increased. A similar trend was observed in the fluorescence spectra (Figure 2b).

In contrast, when the same procedure was performed without any capping agent, the absorbance occurred at wavelengths above 420 nm (Figure 2c), which corresponds to bulk CdS.^20^ This wavelength continued to increase to 450 nm, as more Cd^2+^ was introduced, indicating, as expected, that without a capping agent, the size of the CdS precipitate was not controlled.

After confirming that protein NMB25 capped the growth of quantum dots, we asked whether different *de novo* proteins might produce nanoparticles with different optical properties and/or morphologies. Therefore, we compared NMB25 to three additional *de novo* proteins: NMB20, S824-GGC, and SynI3. All three were effective in capping CdS quantum dots, as demonstrated by the absorbance spectra in Figure 3a. The absorbance of bulk CdS, synthesized without any capping agent, is included for comparison. All four *de novo* proteins capped CdS quantum dots and gave rise to absorbance peaks that are blue-shifted relative to those of bulk CdS, thereby demonstrating quantum confinement due to small nanocrystal size (Figure 3a). For each *de novo* protein, the peak positions occurred at a different wavelength. The sizes of CdS quantum dots can be estimated from these wavelengths using the sizing curves presented by Yu et al.^20^ CdS quantum dots capped by NMB25 or S824-GGC absorb at 340 nm, corresponding to an estimated size of ∼2 nm. In contrast, NMB20 and SynI3 led to absorbance at 360 nm, suggesting particles of ∼2.4 nm, ∼20% larger than the previous examples.

**Figure 3:**
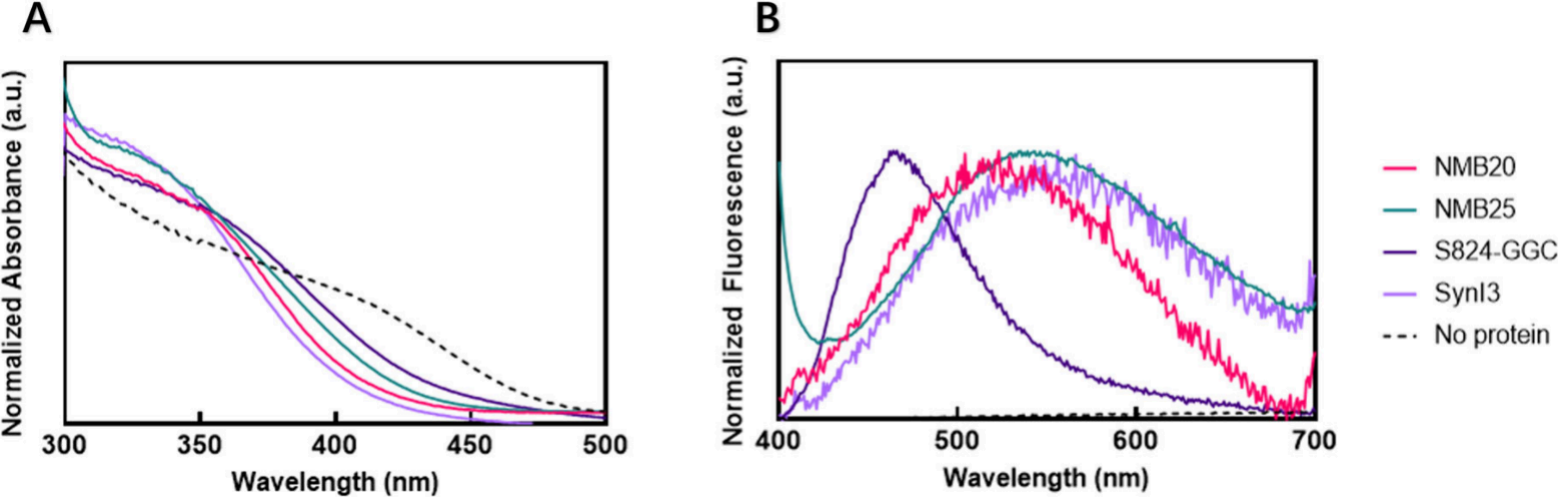
(a) Steady-state absorbance spectra for CdS synthesized in the presence of four different *de novo* proteins as capping agents. (b) Fluorescence emission spectra for nanoparticles templated by these proteins. The dotted line represents the negative control sample synthesized without any protein.

The optical difference between CdS quantum dots capped by the different proteins is even more evident in the normalized fluorescence spectra shown in Figure 3b. CdS nanoparticles synthesized with all four proteins are fluorescent, yet they emit at different wavelengths. NMB20 led to emission at 530 nm, NMB25 at 540 nm, and SynI3 at 550 nm. Nanoparticles capped by S824-GGC, the sole protein containing cysteine, were blue-shifted to 480 nm (Figure 3b). In addition, the samples have different peak widths and shapes, with nanoparticles capped by S824-GGC showing the narrowest peak. As a control, CdS synthesized without any capping agent did not produce measurable fluorescence.

In addition to measuring the optical properties, we used scanning transmission electron microscopy (STEM) to assess the shape and confirm the diameters of the protein-capped materials. As shown in Figure 4a, quantum dots capped by NMB25 appeared as the expected spherical nanoparticles. The average particle size is estimated to be ∼2 nm, with a size variance of ∼0.3 nm and some irregularly shaped particles. This measurement from TEM images aligns with the ∼2 nm size estimated from absorbance wavelength. Lattice fitting was performed on selected nanoparticles capped by NMB25 (Figure 4b), revealing a structure that matches both wurtzite and zinc blende, suggesting a mixed-phase composition. The quantum dots capped by SynI3 (Figure 4c) appeared slightly larger (2.4–2.7 nm), consistent with size estimates from absorbance wavelength measurements. Interestingly, the protein SynI3 occasionally led to a unique assembly of nanorods (Figure 4d, Figure S1), alongside spherical type of quantum dots. These nanorods display a length of approximately 500 nm, and individual spherical particles are discernible at the tips of these nanorods (Figure S1). The composition of these nanorods was assessed using energy-dispersive X-ray spectroscopy (EDS), which confirmed the presence of Cd and S (Figure S2). Additionally, elemental mapping for Cd and S reveals a distinct overlap of these two elements, as shown in Figure 4e and f. The shape of these nanorods could suggest either a head-to-tail assembly of protein-capped CdS quantum dots, or the attachment of CdS quantum dots to a preassembled protein aggregate that serves as a structural backbone to guide the supramolecular arrangement.

**Figure 4:**
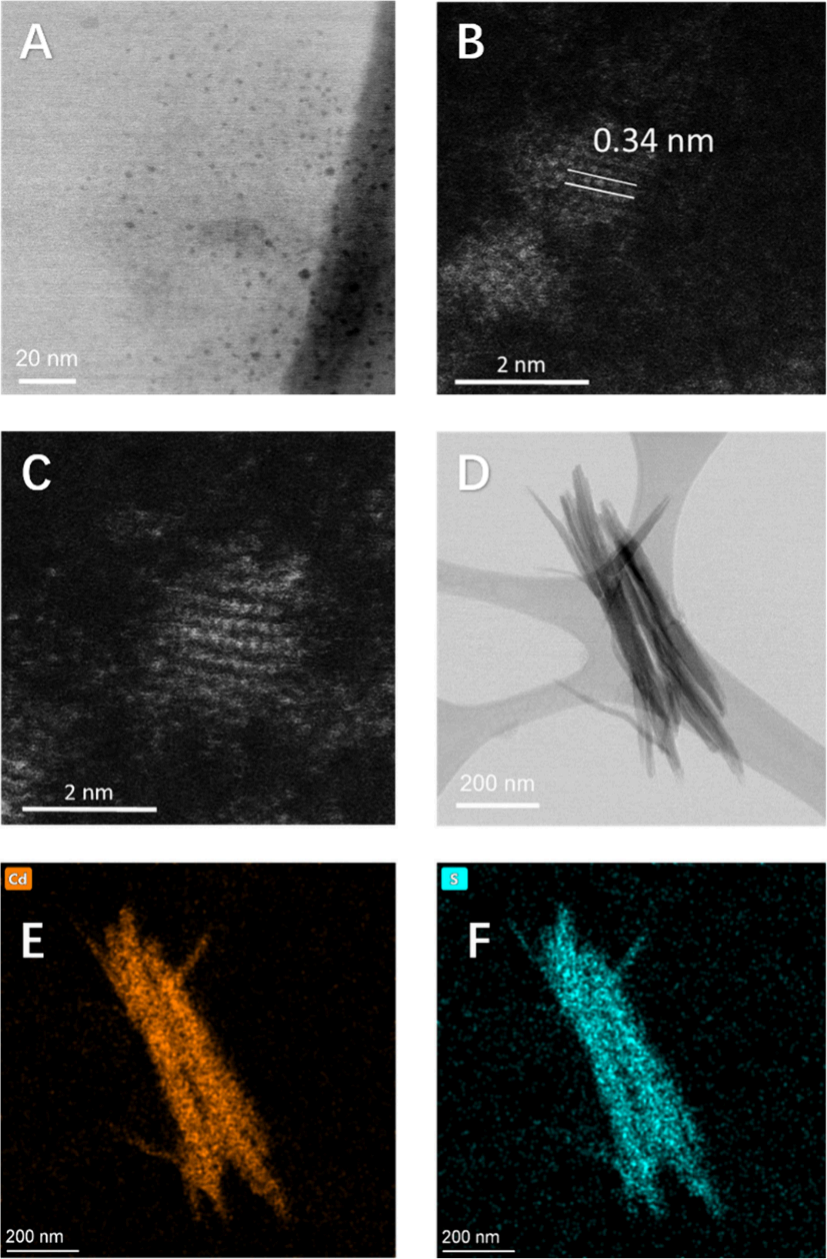
STEM images of protein-capped quantum dots. (a) Bright-field STEM image of CdS quantum dots capped by NMB25 are spherical and separate. (b) High-magnification image of a representative particle that is capped by NMB25 with lattice fitting measurement. (c) CdS quantum dots capped by SynI3 are slightly larger. (d) BF-STEM image of the nanorods. (e) Cd and (f) S elemental maps for the nanorods in (d).

The stability of quantum dots is important for downstream applications, and nanoparticle durability is a key topic in the field.^21,22^ To assess stability over time, we monitored the absorbance of CdS nanoparticles for 7 days at 4 °C (Figure 5a). When S824-GGC was used as a capping agent, the absorbance intensity of the quantum dots decreased slightly, indicating the concentration of nanoparticles might have decreased (Figure 5a). Notably, however, the wavelength of the peaks remained constant at 360 nm. In fluorescence spectra, the wavelength of maximal emission also remained unchanged (Figure 5b). These data indicate that the size of the nanoparticles capped by protein S824-GGC remains constant over time. In contrast, this was not the case for quantum dots capped by l-cysteine, where absorbance red-shifted over time (Figure 5c). The instability of quantum dots capped by l-cysteine was even more notable in the fluorescence spectra, as the peak wavelengths continued to decrease over time (Figure 5d).

**Figure 5:**
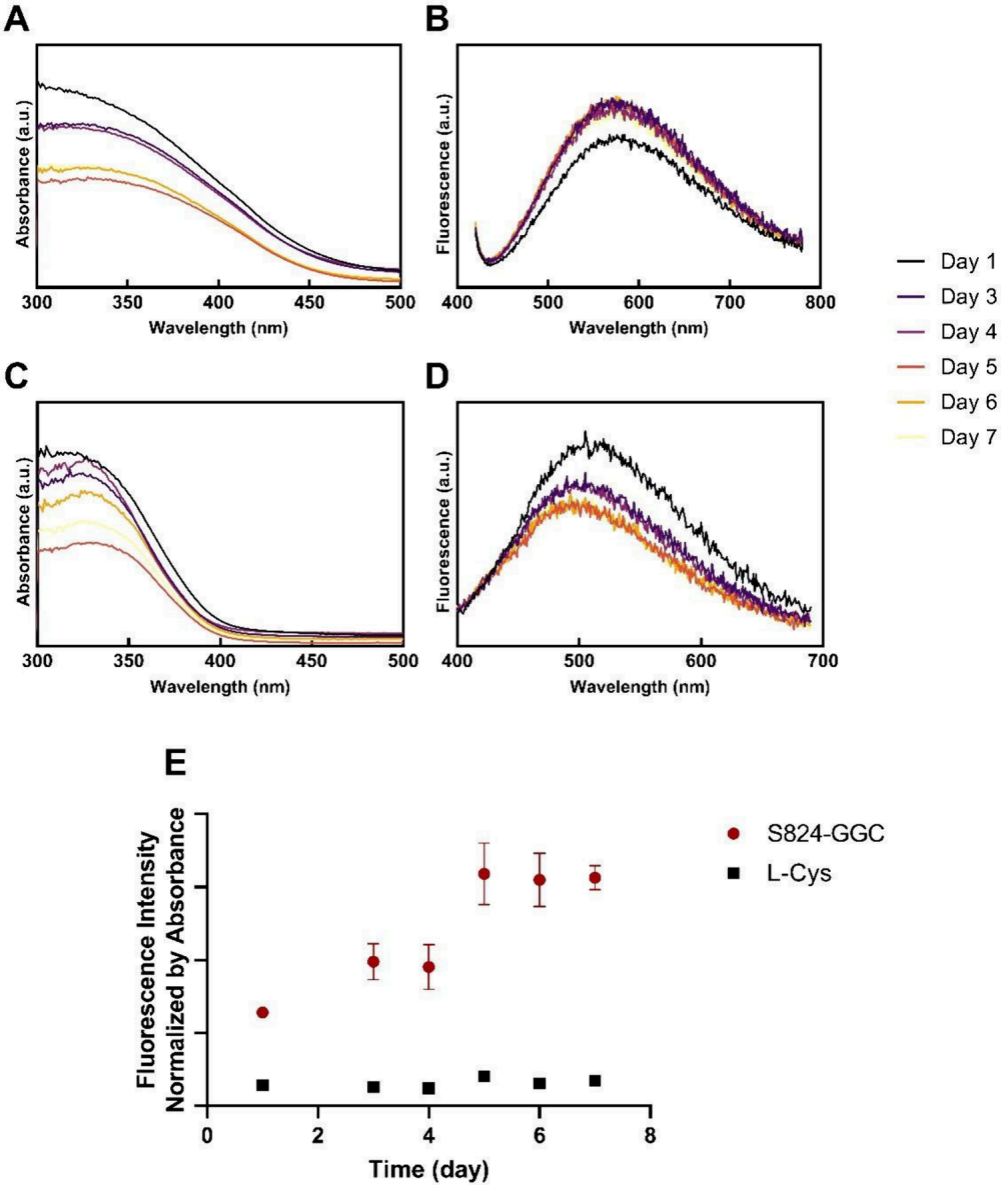
(a) Absorbance and (b) fluorescence spectra show that S824-GGC stabilizes quantum dots over 7 days. (c) Absorbance and (d) fluorescence spectra show that quantum dots capped by l-cysteine are less stable when compared to quantum dots capped by proteins S824-GGC. (e) Max fluorescence intensities for quantum dots capped by S824-GGC and l-cysteine over 7 days after normalizing by absorbance intensities.

In addition to the absorbance and emission wavelengths, the intensities of the fluorescence also suggest that capping by *de novo* proteins may have advantages over small molecules. Despite the possible decrease in particle concentration over time, the emission intensity for quantum dots capped by S824-GGC increased from day 1 to day 3 and remained constant during the next few days. After normalizing for concentration by their absorbance at the peak wavelength, fluorescence intensities obtained from the protein-capped quantum dots were significantly higher than those of quantum dots capped by l-cysteine. Moreover, fluorescence intensity increased over time for the protein-capped sample, while it remained unchanged for capping by l-cysteine (Figure 5e).

When developing technologies for the biogenic production of quantum dots, it is important to also consider the inherent stability of the proteins used for capping. Natural bacterial proteins have an average half-life *in vivo* of ∼20 h.^23,24^ Even *in vitro*, where cellular degradation mechanisms are absent, many proteins are prone to denaturation and/or loss of function. The inherent instability of many natural proteins could pose challenges for their use in the fabrication of protein-coated nanomaterials.

In contrast to natural proteins, *de novo* proteins can, in principle, be engineered for any level of stability. Indeed, previous work in our group has demonstrated that many of our binary patterned *de novo* proteins are resistant to both high temperature and denaturing agents.^40^ Therefore, we hypothesized that our *de novo* proteins might continue to function as capping agents even after the quantum dots have been stored for longer times.

To assess the stability of S824-GGC over time, we measured its secondary structure by circular dichroism (CD) spectroscopy. The CD spectrum of S824-GGC displays two minima at 208 and 220 nm (Figure S3), typical of α-helical proteins. This is consistent with expectations from their binary patterned design, and was observed previously for other proteins from these libraries. Over 7 days, the absolute value of ellipticity decreased slightly, yet S824-GGC remained mostly folded and helical after 7 days (Figure S3). Thus, the superior stability of the protein-capped nanoparticles over time correlates with the inherent stability of protein S824-GGC.

In comparison, cysteine is unstable in water and undergoes rapid oxidation to form the disulfide bonded cystine dimer. In Tris buffer near neutral pH, the half-life of cysteine is estimated to be only 0.1 h.^25^ Therefore, over the course of our 7-day experiment, cystine would become the dominant species. Although the disulfide bonded cystine dimer could potentially interact with metal ions,^26,27^ the poor solubility of cystine in water limits the effective capping ability of cystine.

In contrast to free cysteine, disulfide formation by Cys residues in proteins depends on factors beyond concentration, pH, and temperature. In particular, the proximity to redox-active metal ions^25^ and the local 3-dimensional structure around the Cys residue can exert substantial impacts. Indeed, the Cys residue in protein S824-GGC remains mostly reduced under the conditions of our studies. Thus, the superior capping behavior of protein S824-GGC relative to cysteine may result from the enhanced ability of the protein to remain in the reduced state.

Because the proteins used in this study were derived from combinatorial libraries, they have different amino acid sequences. Nonetheless, all of them successfully capped the growth of CdS quantum dots. We hypothesized that despite their different sequences, all these proteins would have an affinity for Cd^2+^ arising from the abundance of metal binding residues (particularly histidine) in our library design. To investigate this hypothesis, we used isothermal titration calorimetry (ITC) to measure the interaction between each protein and Cd^2+^. The sample cell was filled with purified protein (15**–**20 μM, or 0.125**–**0.25 mg/mL) in Tris buffer, while the syringe contained Cd^2+^ (100**–**500 μM) dissolved in the same buffer. The enthalpies associated with Cd-binding were measured as the heat required to maintain the chamber at constant temperature during a series of 18 injections. Consistent with our hypothesis, all of the proteins that capped nanoparticle growth also bind Cd^2+^. This is observed with the initial significant ΔH, which gradually approaches a plateau through the titration process (Figure 6). Negative ΔH values were recorded for all four proteins, thereby demonstrating exothermic binding to Cd^2+^ (Table S1).

**Figure 6:**
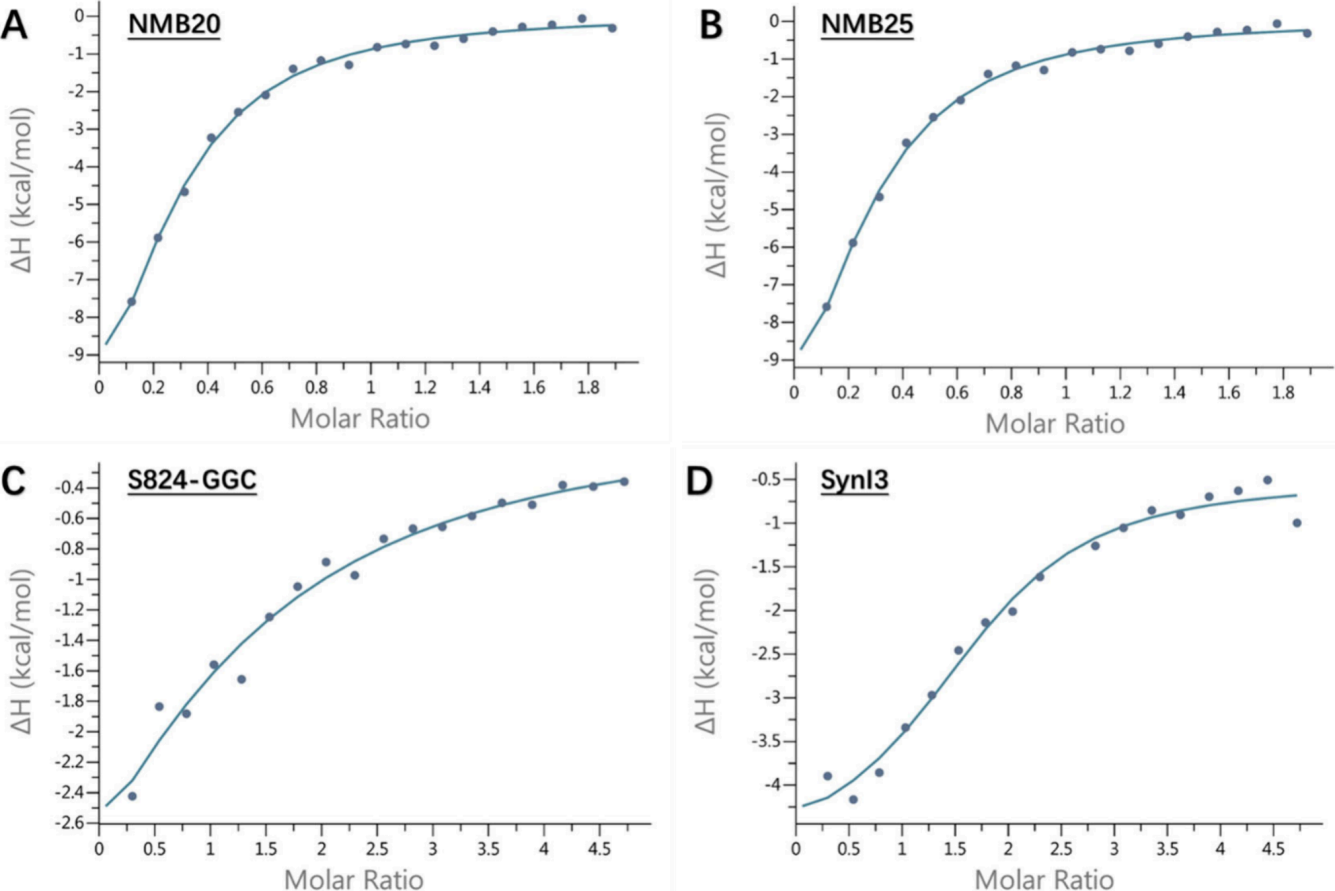
ITC binding curves for interactions between Cd^2+^ and (a) NMB20, (b) NMB25, (c) S824-GGC, and (d) SynI3. Binding parameters were determined by fitting the changes in enthalpy observed during the titrations, assuming a single set of binding sites.

Binding affinities can be estimated from ITC data. Because the proteins in this study were neither evolved nor rationally designed to bind metals, we anticipated they would exhibit low affinities for Cd^2+^, multiple binding sites, and/or poorly defined binding sites. Indeed, our previous work on a larger collection of NMB proteins showed that many of them contained multiple binding sites, and/or binding sites shared between several protein monomers.^9^ Therefore, in that earlier work, we reported binding parameters as average values for all sites on a protein, and represented the affinities as apparent dissociation constants. The K_app_ values for the four proteins in the current study are approximately 4.7 μM for NMB20, 3.8 μM for NMB25, 38 μM for S824-GGC, and 5.5 μM for SynI3 (Table 1). Although cysteine is often associated with metal binding (*k*_app_ ∼ 1.2 μM^28^), S824-GGC does not exhibit a higher affinity, as indicated by its *k*_app_. This may be because the cysteine is located at the C-terminus rather than within a defined binding pocket, making metal coordination less favorable. Additionally, unlike the NMBs, the parental protein, S824, was not explicitly selected for metal binding. Although these values range by a factor of 10, this variance in affinity did not seem to have a major impact on a protein’s ability to cap CdS quantum dots.

**Table 1:** A Summary of Properties of *De Novo* Protein Capping Agents and Their Corresponding QD Products

Protein	Number of Positively Charged Residues	Number of Histidines	Affinity for Cd^2+^, K_app_ (μM)	Optimal Protein Concentration (mg/mL)	Nanocrystal Diameter (nm)	Absorption Maxima (nm)	Photoluminescence Maxima (nm)	Stokes Shift (eV)	Quantum Yield (%)	QY Std. Dev. (%)
**NMB20**	11	13	4.7	1.3	2.4	360	530	1.1	7	0.4
**NMB25**	7	15	3.8	0.88	2	340	540	1.4	9	0.7
**S824-GGC**	9	12	38	0.62	2	340	480	1.1	9	0.8
**SynI3**	14	14	5.5	0.75	2.4	360	550	1.2	12	2

Finally, to confirm the relation between metal binding histidine residues and quantum dot capping, we studied HisZero, a protein devoid of histidine residues. This variant was created using the Rosetta Sequence Tolerance protocol^29^ to replace all histidine residues found in S824-GGC, and it also lacks the Gly-Gly-Cys tail. (A sequence comparison between HisZero and S824-GGC is shown in Figure S4a.) HisZero demonstrated weakened binding toward transition metal ions,^9^ and as expected, it failed to cap the formation of CdS quantum dots, as the resulting material does not emit detectable fluorescence (Figure S4b). This supports the hypothesis that metal binding is the foundation of our proteins’ ability to cap quantum dots.

In recent years, biomineralization has garnered attention as an alternative route to nanoparticle synthesis. This method facilitates the formation of nanoparticles in the aqueous phase, under atmospheric conditions, and at lower temperatures compared to traditional synthesis techniques.^2^ Inspired by natural processes, previous studies have explored two routes to harness the power of biological matter for the development of biogenic synthesis of quantum dots: (i) utilizing enzymes to catalyze the formation of metal chalcogenides, and (ii) templating nanoparticle growth with biological macromolecules. While both methods have been demonstrated with naturally occurring proteins, that approach relies on the discovery of suitable natural proteins, which inherently carry evolutionary baggage and can be challenging to manipulate. In contrast, *de novo* proteins created to fold into specific topologies, offer intrinsically high designability. In particular, proteins from binary patterned libraries enable extensive explorations of sequence space in the context of libraries wherein the identity of amino acids is constrained solely by the polarity/nonpolarity at each residue. This allows for nearly unlimited engineering efforts.

Previously, a *de novo* protein, ConK, was discovered to catalyze the generation of H_2_S, offering a steady flow of reactive sulfur that can be incorporated into the biogenic synthesis of CdS quantum dots.^10^ The results with ConK inspired us to explore if *de novo* proteins can also be employed in the other route of biomineralization, using proteins to template the formation of nanoparticles. The versatility of *de novo* proteins can potentially be beneficial in this direction, as there is evidence that various aspects of peptides, including both the sequence and the conformation, can affect the properties of the resulting quantum dots.^17,18^ In the context of binary patterned 4-helix bundles, the sequence can be varied without affecting the overall topology, thereby enabling the separate study of these two characteristics.

In the current study, we describe the first-ever attempt to employ *de novo* proteins to template the formation of metal sulfide quantum dots. Our findings show that *de novo* proteins clearly demonstrate an ability to precisely control the size of CdS nanoparticles, even as the concentration of CdS continues to increase. To the best of our knowledge, this is the first report of *de novo* proteins exhibiting such behavior. The NMBs and SynI3 were not specifically designed to interact with metal chalcogenides; rather, they were generated using semirandom sequences intended to fold into a 4-helix bundle. Nevertheless, these proteins contain multiple histidines, which are known to be important for metal binding, and numerous charged amino acids located on the protein’s surface, which may also contribute to their binding to CdS.

In previous work, we identified NMB20 as a strong binder of Co­(II) and Cu­(II), and a weak binder of Zn­(II); NMB25 was identified as a moderate binder of Cu­(II), with no observed affinities for Co­(II) or Zn­(II).^9^ These findings suggest that *de novo* proteins exhibit both promiscuity, as evidenced by their ability to bind Cu^2+^, Co^2+^, and Cd^2+^, and selectivity, as they demonstrate a preference for not interacting with Zn^2+^. Future work will explore the behavior of protein capping agents in the synthesis of nanoparticles involving multiple metal ions, such as the growth of a CuS shell onto the NMB25-capped CdS nanoparticles, as it has been shown that CdS/CuS NPs have enhanced photocatalytic activity for hydrogen generation.^30^

In addition to demonstrating the ability of *de novo* proteins to cap the size of CdS quantum dots, we also observed the potential for tuning the optical and physical properties of quantum dots by utilizing different capping proteins. The properties of the *de novo* proteins used as capping agents and the characteristics of the CdS quantum dots made with them, are summarized in Table 1. The sizes of quantum dots synthesized with different *de novo* proteins vary noticeably, as suggested by the different absorption wavelengths. Regarding emission, the utilization of distinct *de novo* protein capping agents results in variations in the emission peak width, with full width at half-maximum (fwhm) values ranging from 75 to 125 nm. Thus, these protein-capped quantum dots may either exhibit a well-defined and sharp emission peak or display a broader emission peak. Additionally, the Stokes shift is estimated to vary within the range of 1.1 to 1.4 eV. When compared to CdS nanoparticles capped by cysteine, which typically have a Stokes shift ∼1 eV, NPs capped by *de novo* proteins exhibit somewhat larger average Stokes shifts. These shifts are also generally larger than observed in chemically synthesized CdS quantum dots (<0.1 eV) where emission occurs from the band-edge.^31^ Our protein capped CdS QDs likely emit from a trap state, commonly observed for biomineralized QDs. Trap-state emission typically results from imperfections at the surface of the nanocrystal that cause defect sites.^32^ The photoluminescence quantum yield (QY) of the CdS quantum dots ranged from 7% to 12% with relative errors on the order of 10% of these values (Table 1). Each sample was prepared independently for measurements to ensure replicability. All data points collected fall within 1.4 standard deviations of the mean. The reported QYs are significantly higher than the QY (2.3%) of CdS quantum dots synthesized previously using biomineralization,^33^ despite being slightly lower than the reported QY (19%) for CdS quantum dots synthesized with small molecule capping agents in aqueous condition without postsynthesis processing.^34^ We anticipate that the QY could be enhanced through strategies such as further purification, postsynthesis processing, and core–shell structure development.

The optical properties of NPs are not only related to their sizes but also to their crystal form and 3-dimensional structure.^35^ Thus, morphologies are important both to demonstrate the tunability of biogenic NPs and to optimize the optical performance of these NPs. Recent studies in the field have increasingly recognized the unique advantages of biogenic approaches may have over traditional chemical methods in controlling the morphology of NPs. For instance, facet-specific peptides were used to direct the synthesis of platinum NPs with different shapes.^36^ One of the *de novo* proteins studied here, SynI3, has shown the potential to induce nanorod formation, although the exact mechanism remains unclear. This intriguing phenomenon might be attributed to a linear protein oligomerization process induced by binding to CdS nanoparticles. Although SynI3 is monomeric alone in solution, contact with CdS may lead it to assemble into an organized supramolecular structure. This linear oligomerization process may serve to guide the formation of these distinctive nanorods. Remarkably, this discovery represents the first instance where biogenic nanorods have been synthesized by harnessing the inherent templating abilities of a *de novo* protein. This observation is similar to using M13 viruses displaying short peptides (6**–**10 amino acids), which self-assemble into fibers.^18^ It indicates that biotic methods can offer advantages in achieving specialized NP morphologies, a task that has presented challenges for conventional techniques.

While the correlation between protein sequence and the resulting quantum dot characteristics remains incompletely understood, our findings highlight the crucial role of Cd^2+^ affinity in enabling capping. The observed affinity for Cd^2+^ likely stems from the presence of multiple histidines, charged amino acids, and a cysteine, in the case of S824-GGC. Since HisZero failed to cap, histidine and cysteine likely play a more decisive role than aspartic acid or glutamic acid. This is consistent with previous observations that negatively charged residues do not appear to significantly contribute to the interaction between proteins and metal chalcogenides.^17^ As summarized in Table 1, all four proteins contain multiple histidine residues and positively charged amino acids. This aligns with previous studies that concluded that Lys and Arg residues are essential for templating the formation of metal-containing NPs.^37,38^ However, the affinity for Cd^2+^ does not appear to transfer directly to how well a protein controls the size of CdS quantum dots. Despite having the lowest affinity, S824-GGC produced quantum dots with a smaller size, measuring 2 nm, approximately 17% smaller than those of quantum dots capped by NMB20 and SynI3. This discrepancy suggests that while Cd binding correlates the ability of a protein to cap quantum dots, it is not directly linked to the size of the final material. Other factors, including packing geometry, likely influence their physical and optical properties. One such consideration may be the number of proteins needed to cap each protein, which could be inferred by considering the optimal protein concentration for capping. We found a variation between proteins that was not directly correlated with affinity (Table 1), suggesting that oligomerization or other factors may influence the protein-nanocrystal surface interaction.

Because of their binary code design, the *de novo* proteins investigated in this study share the same topology as 4-helix bundles. However, the formation of organized supramolecular assemblies, in the form of nanorods in the case of SynI3, suggests that the conformation and oligomerization states of protein capping agents also influence the resulting nanoparticles. In the future, the effect of protein conformation on their ability to template will also be explored, encompassing aspects such as packing, protein oligomerization, and the correlation between the shape of the protein and the morphology of the nanoparticles. In addition, further investigation is needed to assess the ease of separating quantum dots from the synthesis mixture. For TEM sample preparation, excess cadmium and sulfur precursors were removed via dialysis or PD-10 column purification. However, the effects of removing protein capping agents after synthesis remain unexplored. Understanding this aspect will be crucial for evaluating the utility of these quantum dots in potential applications.

## Methods

### Plasmids and Strains

Genes encoding the *de novo* proteins were expressed from a p3GLAR vector (a derivative of a pCA24N vector) containing a chloramphenicol (CAM) resistance cassette. Expression is controlled by a T5 promoter and lac operator. Protein was expressed in BL21 *E. coli* cells (New England Biolabs, Ipswich, MA, USA.)

### Protein Expression and Purification

A single colony was grown in a 5 mL starter culture for 12 h at 37 °C in LB supplemented with 30 μg/mL CAM. Then, 1 mL of starter culture was inoculated into 1 L of LB supplemented with 30 μg/mL CAM. Expression was induced at OD600 = 0.4–0.5 by addition of 100 μM IPTG. After induction, bacteria were incubated for 16 h at 18 °C. Following expression, cells were harvested by centrifugation at 4,500 × g and stored at −80 °C for later use.

Immediately before purification, frozen cells were thawed and resuspended in 30 mL of 50 mM Tris and 300 mM NaCl containing 0.5 mg/mL lysozyme, 10% glycerol, and 1% Triton-100 at pH 7.4. The resuspended cells were then lysed on ice using an ultrasonic homogenizer (Fisherbrand Model 505 Sonic Dismembrator) for a total of 3 min 30 s on time (pulse 10 on/50 off) at 20% amplitude.

Protein was then purified in two steps. First, the filtered lysate was run through a HisTrap HP nickel column (GE Healthcare, Chicago, IL, USA) equilibrated with a running buffer containing 50 mM Tris and 300 mM NaCl at pH 7.4. After a wash step using 50 mM imidazole in the running buffer, the protein was eluted in a buffer containing 375 mM imidazole, 50 mM Tris, and 300 mM NaCl at pH 7.4. Despite the lack of a His-tag, the proteins bind to the nickel column, presumably due to the abundance of surface histidines. The only exception is HisZero, which was expressed with a His-tag that was later cleaved with TEV protease. Second, eluates containing the desired protein were further purified on a HiLoad 26/600 Superdex 75 size-exclusion column (GE Healthcare) in a buffer containing 50 mM Tris and 300 mM NaCl at pH 7.4. Eluates were analyzed by SDS-PAGE (Bio-Rad); protein identity and purity were verified by HPLC (Agilent 100 system, Agilent Zorbax 300SB-C18 column) followed by ESI-MS (Agilent 6220 accurate-mass TOF LC-MS).

### Synthesis of Quantum Dots

A precursor solution of 0.1–1 mM NaSH was premixed with 0–1.5 mg/mL purified *de novo* proteins in 50 mM Tris and 300 mM NaCl at pH 7.4. To this solution, 0.1–1 mM cadmium acetate (99%, Sigma-Aldrich) solution was added while mixing vigorously to form CdS quantum dots capped with proteins. To compare the capping abilities of these proteins to capping by small molecules or uncapped CdS, 0.5 mM cadmium acetate was also added to 0.5 mM NaSH solution preincubated with 10 mM l-cysteine or without any capping agents.

To determine the optimal concentration for each protein capping agent, seven different concentrations (120–130 μM, 95–105 μM, 80 μM, 60 μM, 40 μM, 20 μM, and 10 μM) of each protein were tested.

### Characterization of Quantum Dots

Absorbance spectra were collected on a Cary 6000i UV/vis spectrometer with an integrating sphere attachment (Agilent) to reduce scattering noise. Fluorescence spectra were collected on a QuantaMaster (Horiba) using an excitation wavelength of 340–360 nm.

Absolute quantum yield measurements were performed on a QuantaMaster (Horiba) with an integrating sphere. By measuring the emission spectra of each sample and blank from 355 to 405 nm across the excitation wavelength at 380 nm, absorbed photons were calculated as the difference between the integrated scattering signals of the blank and the sample. By measuring the emission spectra of each sample and blank from 410 to 730 nm, emitted photons were calculated as the difference between the integrated emission of the sample and the blank. The step size was set to 0.25 nm for all measurements. Absolute quantum yield for each sample was determined from the ratio of emitted photons to absorbed photons. Percent error associated with the quantum yield was calculated as the standard deviation of the mean quantum yields obtained from three independently prepared replicate measurements.

High-angle annular dark-field (HAADF) STEM imaging and energy dispersive X-ray spectroscopy (EDX) mappings were performed on a Titan Cubed Themis 300 double Cs-corrected scanning/transmission electron microscope (S/TEM), equipped with an extreme field emission gun source and a super-X EDS system. The system was operated at 200 kV.

### Protein Stability

The stability of the protein samples, as well as the proteins mixed with cadmium, was assessed using a circular dichroism (CD) spectrometer (Chirascan V100). Samples were 15 μM protein in 50 mM Tris and 300 mM NaCl at pH 7.4. The protein with cadmium samples were made with 15 μM protein and 0.25 mM cadmium acetate in 50 mM Tris and 300 mM NaCl at pH 7.4. All samples were stored at 4 °C and measured by CD daily for 7 days. The wavelength range for all samples was from 197 to 260 nm.

### Isothermal Titration Calorimetry (ITC)

The affinities of proteins for Cd^2+^ were assessed on a MicroCal PEAQ-ITC calorimeter (Malvern Panalytical). Titrations were performed at 25 °C in a cell containing 300 μL of protein (15 or 20 μM) in 50 mM Tris and 300 mM NaCl at pH 7.4. The syringe was filled with 200 or 500 μM cadmium acetate solution in the same buffer. Each titration consisted of 18 injections of 2 μL with an 8-s duration and a 120-s interval between injections. For all experiments, a reference power of 10 μcal, high feedback mode, and a stirring speed of 750 rpm were used. The energies required to hold the temperature constant were analyzed by the MicroCal PEAQ-ITC software to estimate the binding enthalpy.

## Supplementary Material



## Notes

Additional TEM images and EDS spectrum obtained for the protein-capped quantum dots, additional CD spectra for the stability studies, and additional binding studies (PDF)
