# Supplementary material for: De Novo Proteins Template the Formation of Semiconductor Quantum Dots
Source: ACS Cent Sci. 2025 May 27;11(6):983–93. doi: 10.1021/acscentsci.4c01826 (PMC12220126; doi:10.1021/acscentsci.4c01826)
Supplement: Supplementary file 1 [file oc4c01826_si_001.pdf]

Supplemental Information

De Novo Proteins Template the Formation of Semiconductor Quantum Dots

Yueyu Yao[1], Jingyun Wu[1], Yue Hu[1], Laura Haubold[2], Obinna Uzosike[2], Guangming Cheng[3], Nan Yao[3], Gregory Scholes[1], Michael H. Hecht[1, 2]* and Leah Spangler[4]*

[1] Department of Chemistry, [2] Department of Molecular Biology, [3] Princeton Materials Institute, Princeton University, Princeton, NJ 08544, USA.

[4] Department of Chemical and Life Science Engineering, Virginia Commonwealth University, Richmond, VA 23284, USA.

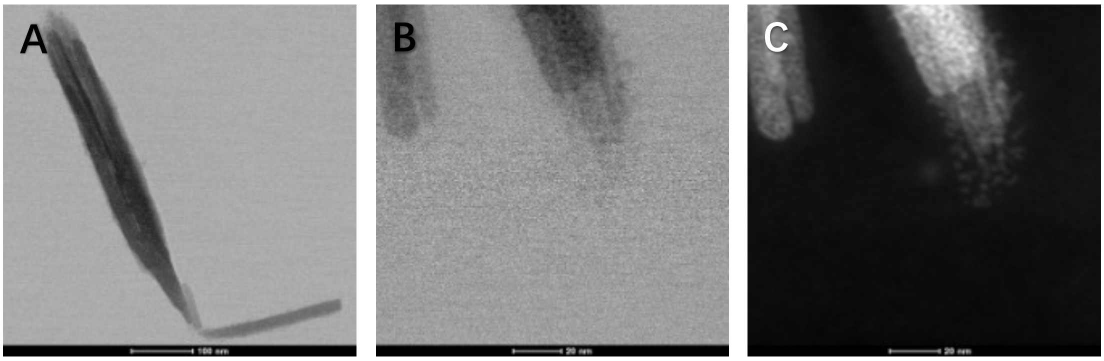

Figure S1. Additional TEM images of CdS quantum dots synthesized with SynI3 as the capping agents. The nanorod-shaped morphology is the dominant species observed on the grid. At the tip of each fiber, individual nanocrystals can be seen.

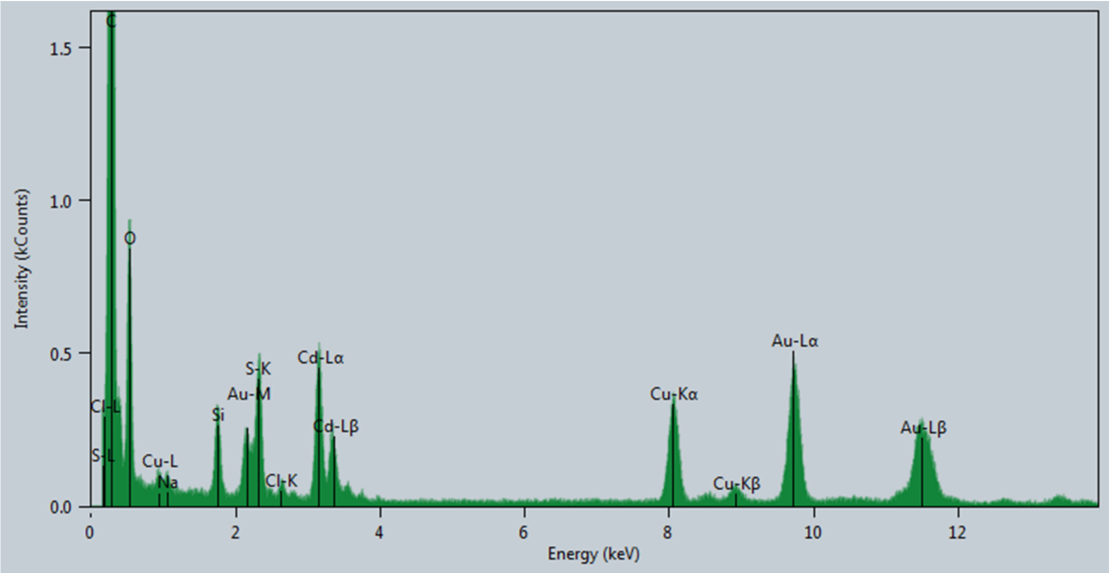

Figure S2. EDS spectrum obtained for the nanorods shows clear peaks corresponding to Cd and S. Peaks corresponding to Au and Cu are artifacts from the grid and TEM instrument.

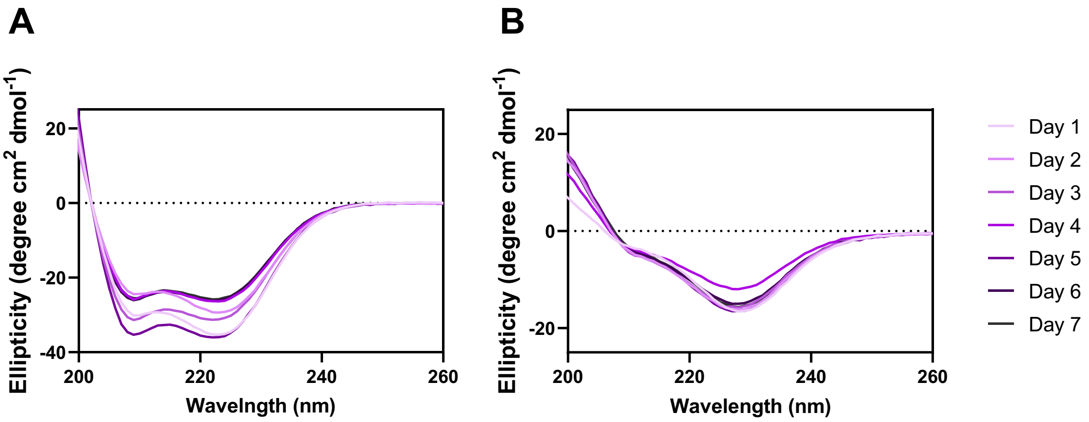

Figure S3. CD spectra over seven days for (a) S824-GGC and (b) S824-GGC with 0.5 mM Cd^{2+}. Measurements were performed in duplicates.

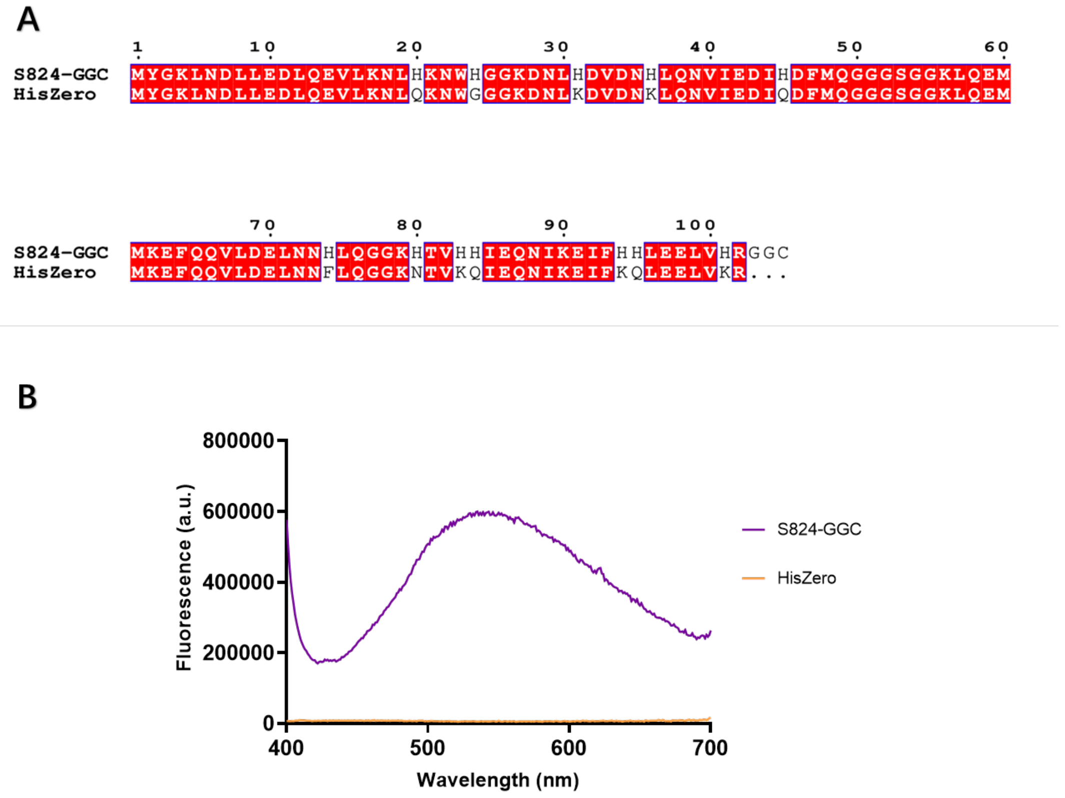

Figure S4. (a) Sequence comparison between S824-GGC and HisZero. All histidine residues on S824-GGC along with the cysteine-containing tail were removed to construct HisZero. (b) HisZero failed to produce fluorescent material, in contrast to S824-GGC.

Table S1. ITC binding parameters for *de novo* protein capping agents.

Protein	*K_d (μM)*	*ΔH (kcal/mol)*	*ΔG (kcal/mol)*
NMB20	4.76 ± 1.18	-18.1 ± 4.44	-7.26
NMB25	3.82 ± 1.10	-11.7 ± 1.62	-7.39
S824-GGC	38.0 ± 47.8	-7.06 ± 10.5	-6.03
SynI3	5.53 ± 2.32	-4.37 ± 0.540	-7.17
